# De Barsy Syndrome: A Case Report of a Rare Genetic Disorder

**DOI:** 10.7759/cureus.33280

**Published:** 2023-01-02

**Authors:** Kankipati Srimeghana, Saikrishna Dodda, Anagha SK, Tamara Tango, Aishwar Dixit, Sweta Sahu

**Affiliations:** 1 Medicine and Surgery, Andhra Medical College, Visakhapatnam, IND; 2 Medicine, Navodaya Medical College, Raichur, IND; 3 Internal Medicine, Government Medical College Thiruvananthapuram, Thiruvananthapuram, IND; 4 Pediatrics, Faculty of Medicine Universitas Indonesia, Jakarta, IDN; 5 Internal Medicine, Baba Raghav Das Medical College, Gorakhpur , IND; 6 Surgery, Jagadguru Jayadeva Murugarajendra (JJM) Medical College, Davanagere, IND

**Keywords:** rare genetic disorder, progeroid feature, congenital cutis laxa, rare autosomal recessive disorder, de barsy syndrome

## Abstract

De Barsy syndrome (DBS) is an exceedingly rare autosomal recessively inherited genetic disorder that manifests as premature aging with progeroid features. Typically, the skin loses its elasticity, causing laxity, wrinkling, and sagging. Other characteristics include ophthalmological, orthopedic, and neurological abnormalities. As of 2011, only 27 DBS cases had been recorded. This paper reports the case of a two-day-old female infant who was referred to the pediatrics department with complaints of lax skin, a progeroid appearance, a short stature, hazy corneas, and bilateral claw-like hands with thin overlapping fingers. She also had features of pectus excavatum and visible veins over her chest and abdomen. There was a history of third-degree consanguineous parents in this patient. This patient was diagnosed with De Barsy syndrome due to findings on the Verhoeff Van Gieson staining, which demonstrated a marked decrease in elastic tissue fibers. Palliative care was recommended for this infant. We report this case considering its extreme rarity.

## Introduction

Dr. De Barsy first described the de Barsy syndrome (DBS) in 1967 as an autosomal recessive disorder with a characteristic prematurely aged appearance (progeroid features), facial dysmorphism, and cutis laxa type III (redundant inelastic skin). Other significant findings of this condition include ocular abnormalities (corneal clouding), musculoskeletal abnormalities, growth retardation, and intellectual disability [1-2]. The prevalence of the disease is unknown, but the National Organization for Rare Disorders (NORDs) reported less than 50 cases in the world literature to date, which makes this disease a highly uncommon genetic disorder. Mutations in the ALDH18A1 gene or PYCR1 genes have been associated with de Barsy syndrome in a few cases, though there is no concrete evidence linking a specific gene to the syndrome [3]. The history of the documented cases revealed that siblings of the same family were affected, suggesting an autosomal recessive mode of inheritance [4]. Our case report presents an infant who was discovered to have the classical presentation of de Barsy syndrome.

## Case presentation

**Figure 1:**
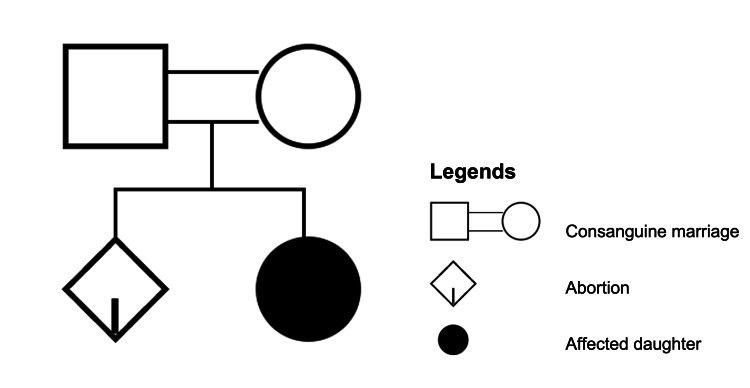
History of consanguine marriage in the family

A two-day-old female neonate was referred to the pediatrics department with the mother of the baby complaining of laxity of skin, thin body stature, a progeroid feature, bilateral claw-like hands, and mild respiratory distress. This female infant was the first live child born to healthy parents with a history of third-degree consanguinity (Figure 1) of marriage and without a significant history of congenital abnormalities in the family.

The mother, aged 22 years, had regular ante-natal check-ups, consisting of three doctor visits in the first trimester and six doctor visits in the third trimester. There was no history of fever or rash in the first trimester. She was not on any medication for other conditions. Her thyroid function tests were normal. Iron and folic acid (IFA) prophylaxis and two doses of tetanus toxoid were administered in the second trimester. The mother had a history of first-trimester abortion one year ago. There was no history of anemia, pregnancy-induced hypertension (PIH), or exposure to radiation or chemicals during pregnancy.

Intrauterine growth retardation (IUGR) was noted in the third trimester. The mother underwent a normal vaginal delivery at full term of gestational age. The child did not cry immediately after birth. The infant’s birth weight was 1500 g, birth length was 44 cm, head circumference was 30.8 cm, chest circumference was 29.7 cm, and ponderal index was 1.88.

**Figure 2:**
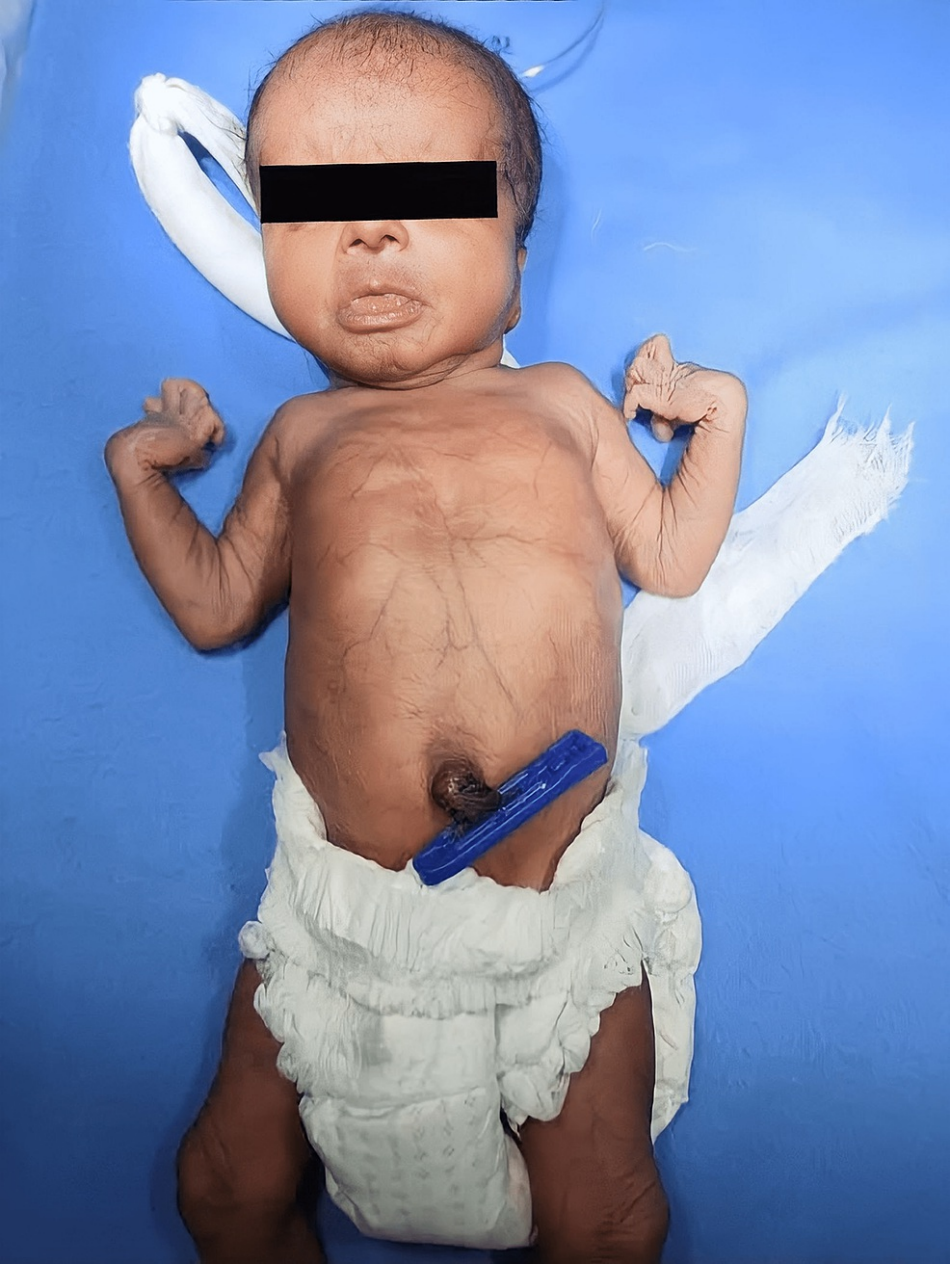
A two-day-old female neonate with lax skin, thin body stature, progeroid appearance, bilateral claw-like hands, and visible veins over her chest and abdomen

The vital signs of the patient were within normal limits. The appearance of the child is progeroid with frontal bossing, dysmorphic facies, micropthalmia with strabismus, and a long philtrum (Figure 2). The anterior and posterior fontanels were wide. The ears were low-set with large ear lobes. There were excessive skin folds over the limbs and generalized hypotonia. The upper limbs showed flexion deformity of the wrists and hyper-extensible metacarpophalangeal joints associated with clawing of the hands. The chest wall showed marked pectus excavatum. The skin over the abdomen and thorax was very thin and transparent. There were visible veins over the chest and abdomen. Examination of the external genitalia revealed mild clitoromegaly.

**Figure 3:**
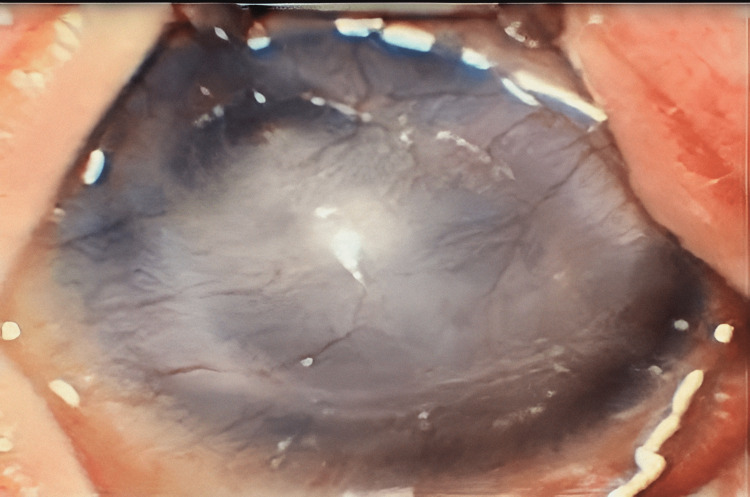
Corneal opacification

Examination of the eyes revealed hypotelorism, bilateral corneal opacities (Figure 3), and cataract formation.

Rooting and sucking reflexes were normal with incomplete Moro’s reflexes. The dermatological evaluation revealed wrinkled, thin, atrophic skin with reduced subcutaneous fat. Verhoeff-van Gieson's staining of the tissue from the skin biopsy demonstrated scanty, tiny, and fragmented elastic fibers, as well as a noticeably reduced amount of elastic tissue fibres in the lower dermis. With the above features, a provisional diagnosis of de Barsy syndrome was established by a dermatologist, endocrinologist, and orthopedic surgeon.

**Figure 4:**
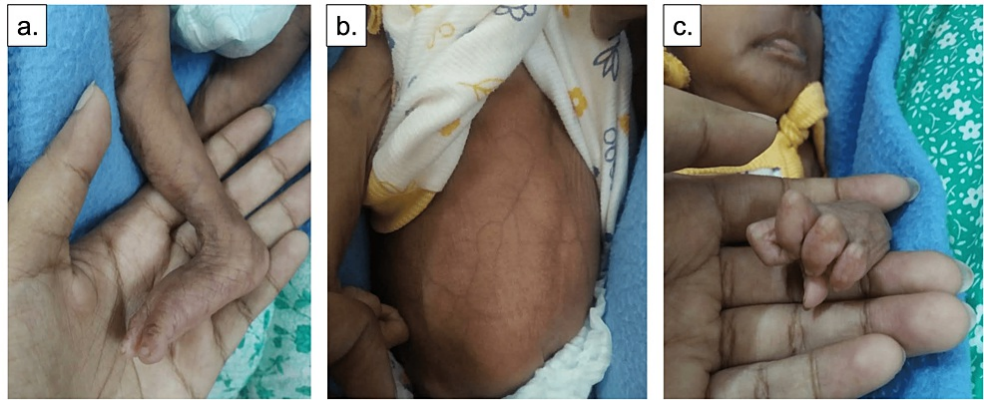
(a) Immobility of right lower limb; (b) visible veins over thorax and abdomen; (c) claw-like hand

The infant was brought to the hospital after one month with complaints of cold, cough, and immobility of the right lower limb (Figure 4a). There was no improvement in this infant’s condition. Corneal opacities became more apparent. The skin folds were also prominent, with visible veins over the thorax and abdomen (Figure 4b). Bilateral clawing of hands was noted (Figure 4c).

The normal temperature was kept to keep the infant warm while monitoring her airway, breathing, and heart rate. This patient was then given 2 litres/minutes of oxygen via nasal prongs for 24 hours, 5 mL cup and spoon feeds. To address the tetany that may have contributed to her hands clawing, she was given an injection of 2 mL of calcium gluconate. Additionally, since the baby was having mild respiratory distress, she received 75 mL of intravenous 10% dextrose at 3 mcL/minute, 25 mg of intravenous amikacin once a day, and 75 mg of intravenous cefotaxime twice a day.

## Discussion

This two-day-old female neonate in our case report was diagnosed with De Barsy syndrome based on the patient's history, clinical manifestations, and supporting examinations. De Barsy syndrome has several alternate names, including de Barsy-Moens-Diercks syndrome, cutis laxa-growth deficit syndrome, and progeroid syndrome of de Barsy [5-6]. Not many cases have been recorded in the medical literature to this day after de Barsy et al. first description in 1968 [1,5,7]. Considering its rarity, we report a case of De Barsy syndrome in a two-day-old female neonate.

Male preponderance has been noted among DBS cases, although there is no distinction between races [1]. DBS is inherited in an autosomal recessive fashion. There is still a probability of additional siblings in the same family being affected [2,4]. De Barsy syndrome can be further categorized into autosomal recessive-cutis laxa type 3A (caused by a mutation in ALDH18A1) and autosomal recessive-cutis laxa type 3B (caused by a mutation in PYCR1) [8-9]. De Barsy syndrome may be caused by a mutation in PYCR1, which codes for a mitochondrial enzyme involved in the metabolism of proline. A study conducted by Leao-Jeles et al. showed that mutations in the ATP6V0A2 gene, also known as ATP6V0A2-CDG under the new naming scheme, may be connected to De Barsy syndrome as well [10]. In this patient, there is a history of consanguineous marriage in this infant’s family, which supports the theory of autosomal recessive as its mode of inheritance.

Most of the affected children (96%) suffer from IUGR, as seen in our patient during the third trimester, and exhibit postnatal growth retardation [1]. Our patient had characteristics of cutis laxa, progeroid appearance, and corneal clouding. Skin abnormalities are one of the clinical manifestations in DBS patients. Cutis laxa in De Barsy syndrome is identified as a cutis laxa type 3 subgroup [5]. Cutis laxa in this patient was observed as loose skin all over her body. In addition to cutis laxa, thin, transparent skin with diminished subcutaneous fat has been described, and 34% of cases have an inguinal and umbilical hernia [1,11]. Moreover, this patient’s face was seen to be prematurely aged in appearance. Generally, patients with DBS have a large forehead, low-set dysplastic ears, sparse hair, a small mouth with thin lips, and a small, turned-up nose, which are progeroid-like traits [1]. Dermal hypoplasia is the cause of the progeroid appearance [4,5,12]. Similar to other progeria-like diseases, Pontz et al. reported decreased fibroblast chemotaxis [1,4]. Immunohistochemistry examination that was carried out on fibroblasts from the DBS patients by Guerra et al. demonstrated more expression of fibrillin-1, normal expression of fibrillin-2, the most expression of tumor necrosis factor, and the least expression of transforming growth factor. They hypothesized that these findings indicated a deteriorating process and a poor rate of elastin synthesis [1,7]. This theory is supported by the result of the Verhoeff Van Gieson staining of this patient, which demonstrated a marked decrease in elastic tissue fibers.

Moreover, corneal clouding is also a characteristic of DBS. Bowman's membrane degeneration causes congenital corneal opacities in 48% of instances. Aldave et al. previously evaluated corneal opacification among DBS patients by utilizing histological examination and electron microscopy. They discovered that the Bowman's layer had lost some of its normal architecture, indicated by a decreased collagen level, tiny bundles of elastic microfibrils in the anterior stroma, and the absence of elastic fibers. Other ophthalmological abnormalities that can be seen among DBS patients include cataracts, myopia, strabismus, and blue sclera, which may appear in variable degrees [13]. Nervous system abnormalities may also be observed among DBS patients. About 76% of individuals have developmental delays since birth, whereas 48% of them have severe delays [1,7,11]. We did not have a long follow-up period for this patient, so we did not know if he had developmental delay, which is a limitation of our study. There have also been reports of skeletal abnormalities among DBS patients, such as hypotonia, athetoid movements, grimacing, and convulsions [1,7,11]. Examples of hyperextensible joints with subluxation or dislocation of smaller joints include hip dysplasia or dislocation, sclerosis, congenital vertical talus, and pectus excavatum [14-15]. In this patient, we observed the presence of hypotonia and pectus excavatum.

There are several differential diagnoses of DBS, including autosomal recessive cutis laxa type 1 (MIM219100), autosomal dominant cutis laxa (MIM123700), and Widemann-Rautenstrauch neonatal progeroid syndrome. To differentiate DBS from these diseases, understanding the mode of inheritance of the disease is important. In addition, autosomal recessive cutis laxa type 1 (MIM219100) is associated with the fibrillin-5 mutation, while DBS is associated with more expression of fibrillin-1 and normal expression of fibrillin-2. Autosomal recessive cutis laxa type 1 (MIM219100) is also associated with diaphragmatic defects and early-onset emphysema [1], while DBS is associated with ophthalmologic defects. Moreover, Widemann-Rautenstrauch neonatal progeroid syndrome shares some characteristics with DBS, such as an elderly appearance from birth, broad cranial sutures and fontanelles, and scanty hair. However, it does not have DBS’s ophthalmological abnormalities or athetoid motions [16-17].

The life expectancy of de Barsy syndrome patients is currently unknown, and further research studies are required to determine mortality rates. A previous study by Kunze et al. in 1985 was the oldest recorded case at the time, in which the patient started walking at the age of four months, uttered his first words when he was nine months old, and did not speak until he was 25 years old. The patient in that case report also had severe developmental delays [2].

## Conclusions

De Barsy syndrome should be suspected in children who have progeroid traits, skeletal abnormalities, or opthalmological abnormalities. Further investigations, such as histological examination of the skin biopsy and genetic studies, are helpful in aiding the diagnosis. However, due to the local unavailability of modern techniques and financial constraints, genetic studies could not be conducted. DBS patients have varying degrees of clinical features. Some pass away in childhood due to severe neurological impairment and ongoing infections. In many individuals with PYCR1 mutations, progeroid traits spontaneously improve, and in some children, the movement disorder remains and does not progress any further. Proper supportive care should be provided to the child, and consultation with the experts is advised as and when required.
